# Insights into the Roles of GLP-1, DPP-4, and SGLT2 at the Crossroads of Cardiovascular, Renal, and Metabolic Pathophysiology

**DOI:** 10.3390/cells14050387

**Published:** 2025-03-06

**Authors:** Melania Gaggini, Laura Sabatino, Adrian Florentin Suman, Kyriazoula Chatzianagnostou, Cristina Vassalle

**Affiliations:** 1 Institute of Clinical Physiology, National Research Council, Via G. Moruzzi 1, 56124 Pisa, Italy; melania.gaggini@cnr.it (M.G.); laura.sabatino@cnr.it (L.S.);; 2 Fondazione CNR-Regione Toscana G Monasterio, Via G. Moruzzi 1, 56124 Pisa, Italy; zulachat@ftgm.it

**Keywords:** type 2 diabetes, glucagon-like peptide 1 agonists, dipeptidyl peptidase-4 inhibitors, sodium–glucose cotransporter 2 inhibitors, cardiorenal system, glycemic control

## Abstract

In recent years, new drugs for the treatment of type 2 diabetes (T2D) have been proposed, including glucagon-like peptide 1 (GLP-1) agonists or sodium–glucose cotransporter 2 (SGLT2) inhibitors and dipeptidyl peptidase-4 (DPP-4) inhibitors. Over time, some of these agents (in particular, GLP-1 agonists and SGLT2 inhibitors), which were initially developed for their glucose-lowering actions, have demonstrated significant beneficial pleiotropic effects, thus expanding their potential therapeutic applications. This review aims to discuss the mechanisms, pleiotropic effects, and therapeutic potential of GLP-1, DPP-4, and SGLT2, with a particular focus on their cardiorenal benefits beyond glycemic control.

## 1. Introduction

In recent years, the rapid and progressive increase in the prevalence of type 2 diabetes (T2D), which is estimated to exceed 693 million by 2045, has highlighted the need for awareness and timely and appropriate treatments to achieve the best control for this condition together with a reduction in complications and risk of cardiovascular (CV) disease, chronic kidney disease, and mortality [1]. Until a few years ago, the management of T2D was essentially based on the achievement of adequate glycemic control, but nowadays, the choice of oral hypoglycemic agent is made according to the individualized risk profile of the patient, considering comorbid factors as well as the risk of hypoglycemia. However, despite these tools, T2D management remains challenging because some diabetic patients have poor optimal glycemic control, which increases the risk of complications, making a profound change in the paradigm of T2D therapy necessary.

In recent years, new drugs for the treatment of T2D have expanded to include glucagon-like peptide 1 receptor agonists (GLP-1RAs), dipeptidyl peptidase 4 inhibitors (DPP-4is), and sodium–glucose cotransporter 2 inhibitors (SGLT2is). These agents (in particular, GLP-1RAs and SGLT2is) were initially developed for their glucose-lowering action but, over time, have been found to demonstrate protective effects toward other therapeutic targets. These new tools profoundly expanded the therapeutic scenario, going beyond glycemic control to promote glucose optimization, while simultaneously improving the effects on weight and hypoglycemia risk, as well as reducing the onset and progression of cardiovascular and renal disease in patients with T2D [2].

In light of these characteristics, ongoing and future studies should continue to better define the role of these drugs in cardiovascular, renal, and metabolic pathophysiology, potentially increasing their applications beyond T2D. However, although SGLT2is and GLP-1RAs, in particular, are recommended in current guidelines for patients with T2D, especially those at high risk for atherosclerosis, there remains an important gap in their use due to their costs and the low rate of prescription by specialists other than diabetologists (e.g., cardiologists and nephrologists), highlighting the need for specific actions by primary healthcare professionals and specialists and greater efforts to improve discussion and knowledge of these drugs to improve T2D management [3,4,5,6].

For this purpose, this review aims to report on the available data on the mechanisms of action and pleiotropic effects of these new T2D drugs (GLP-1RAs, DPP-4is, and SGLT2is) on the cardiorenal system. The authors hope that it may serve as a basis for further discussion in order to expand the possibility of more options for individualized treatments to be applied in the future, which appropriately fit the specific characteristics and needs of each patient.

## 2. GLP-1 and GLP-1RAs

Glucagon-like peptide-1 (GLP-1) is a 30-amino-acid peptide derived from its precursor, proglucagon, a protein expressed in enteroendocrine cells and α cells of the pancreas, as well as in the nucleus of the solitary tract (NTS) in the brainstem. The main sources of GLP-1 in the periphery are epithelial L cells, which are located throughout the intestine and whose density varies along a proximal-to-distal axis, with a limited number of cells in the duodenum, an increasing number in the jejunum, and a greater abundance toward the distal ileum and colon [7]. Peripheral GLP-1 secretion is triggered by carbohydrates, proteins, and fats present in a meal [8] and has the ability to trigger further GLP-1 secretion in the brain. GLP-1 released in the intestine can also act through GLP-1 receptors expressed on the vagus nerve, leading to vagal excitation, which then stimulates NTS preproglucagon neurons, stimulating GLP-1 release in the brain [9]. Through this process, peripheral GLP-1 secretion has a direct effect on central GLP-1 secretion, despite the rapid elimination of the circulating peptide. Interestingly, this connection between peripheral and central GLP-1 secretion may play a significant role in GLP-1 enhancement of the insulin response.

Interest in GLP-1 increased due to the fact that, unlike several other intestinal peptides with insulinotropic effects, it is effective in T2D patients, correcting excessive insulin production. This important aspect led researchers to develop new drugs based on GLP-1, making this compound one of the most investigated of the last few decades. GLP-1 RAs, also known as GLP-1 analogs or incretin mimetics, simulate GLP-1 effects, and, as such, were originally utilized for the treatment of T2D and obesity (improving glucose homeostasis and modulating the sense of appetite, with low risk of hypoglycemia). They also retain beneficial properties for the cardiorenal system; e.g., benefits in endothelial function and recovery from ischemic damage, preservation of myocardial function, reduction in oxidative stress and inflammation, diuretic effect with increased glucose and sodium excretion, and reduction in blood pressure, as well as reduction in intraglomerular pressure and promotion of natriuresis to counter the progression of kidney diseases in T2D patients [8,10].

It has been generally thought that GLP-1 may act as a hormone, using the bloodstream to communicate information over distances [11]. However, several considerations cast doubt on the endocrine mechanism of the GLP-1. In fact, plasma levels of GLP-1 are limited to a very narrow range, even after meal ingestion, despite the highly effective insulinotropic effects of the peptide [12]. Moreover, GLP-1 has a very rapid metabolism, with a half-life of 1–2 min in the plasma of mammals, and is rapidly inactivated mainly by the ubiquitous dipeptidyl peptidase-4 (DDP-4) enzyme and to a lesser degree by other peptidases [13]. Only a minimum amount of secreted GLP-1 reaches the arterial circulation, in contrast to the magnitude of its effects, which is in clear contradiction with a classical endocrine model.

**Figure 1:**
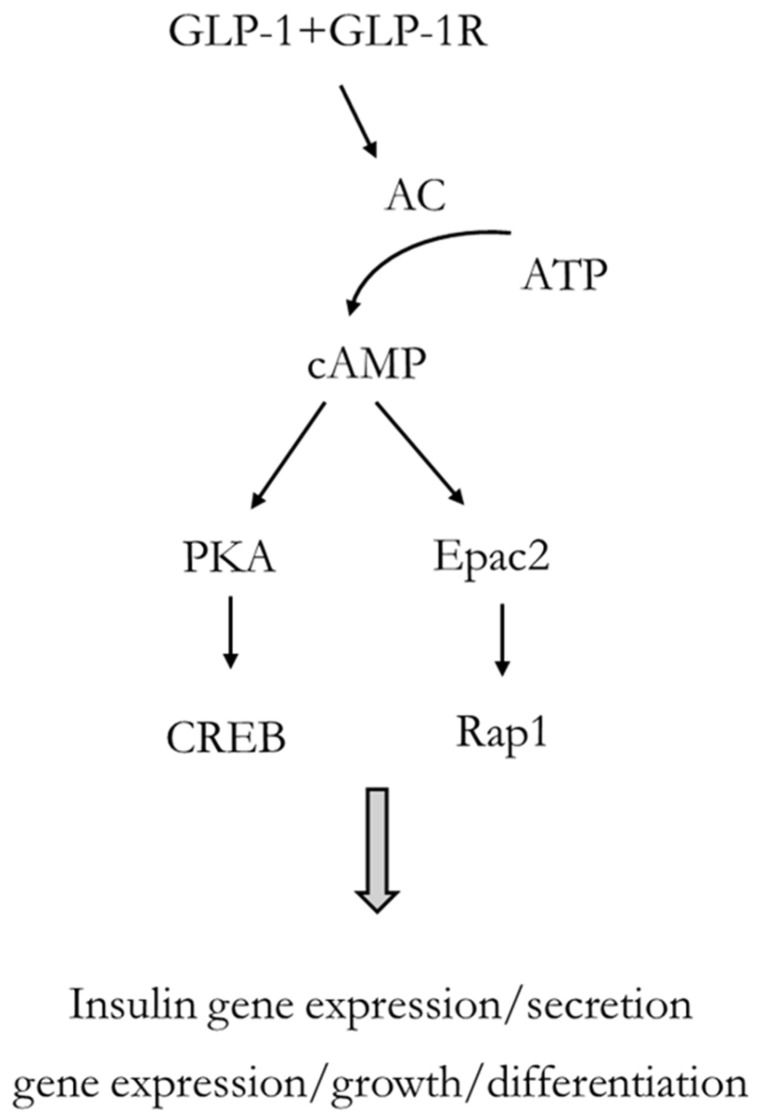
Schematic representation of GLP-1-mediated effects in β cells. For further explanations, please see the text. GLP-1: glucagon-like peptide-1; GLP-1R: glucagon-like peptide-1 receptor; AC: adenyl cyclase; cAMP: cyclic adenosine monophosphate; ATP adenosine triphosphate; PKA: protein kinase A; Epac2: cAMP-regulated guanine nucleotide exchange factor; Rap1: Ras-related protein 1; CREB: cAMP-responsive element binding.

GLP-1 acts through a specific GLP-1 receptor (GLP-1R), principally expressed by β cells of pancreatic islets but present also in many other target tissues, such as lung, stomach, intestine, kidney, heart, and brain tissues [14,15]. GLP-1 stimulates glucose-dependent insulin secretion by pancreatic β cells, inhibits glucagon secretion, and regulates postprandial glycemia, as well as cytoprotective and anti-inflammatory activities on β cells [16,17,18]. Such beneficial effects in blood glucose lowering indicated GLP-1 as a crucial therapeutic target for the treatment of T2D and strengthened the need to define the molecular basis of GLP-1/GLP1R-mediated signaling. Upon binding to GLP-1, GLP-1R initiates a cascade of events, starting from activation of adenyl cyclase and cyclic adenosine monophosphate (cAMP) production, which rapidly potentiates glucose-dependent insulin release. Downstream to cAMP signaling, intracellular activation of protein kinase A (PKA) and cAMP-regulated guanine nucleotide exchange factor (cAMP-GEFII or Epac2) are considered the main pathways mediating the metabolic effects of GLP-1 (Figure 1). The Epac2-dependent action is more important for the priming process, whereas the PKA-dependent mechanism involves the physical recruitment of granules from the reserve pool [19]. The two cAMP-signaling pathways also differ in their dependence on concentration; in fact, the PKA-independent pathway needs a 5-fold higher cAMP concentration to be activated with respect to the PKA-dependent pathway [20]. The activation of these two cAMP-dependent pathways allows GLP-1 to initiate a wide variety of mechanisms within the cell to ultimately trigger insulin release.

The beneficial effects of GLP-1 result from the cAMP-activated PKA signaling in the activation of pro-survival cAMP-responsive element binding (CREB) which, upon phosphorylation at Ser133, promotes its interaction with target genes containing palindromic CRE repeat sequences, including the same insulin gene [21]. Epac2 couples cAMP production to the activation of Rap1, a Ras-related GTPase that plays an important role in the control of Ca^2+^-dependent exocytosis, in β cells and in other cell types [22]. In fact, the Epac family are cAMP-binding proteins, able to stimulate the exchange of GDP for GTP at the guanine nucleotide-binding site of Rap1 [23,24]. In the absence of cAMP, the regulatory region of Epac2 is responsible for the autoinhibition of the catalytic function. The lower-affinity cAMP-binding domain does not play any role in the cAMP-dependent disinhibition of Epac2 guanyl nucleotide exchange activity. However, it is reported to play a role in plasma membrane targeting of Epac2 within β cells [25].

## 3. GLP-1RAs

GLP-1RAs include different molecules, derived by the modification of either native human GLP-1 (liraglutide, semaglutide, dulaglutide, and albiglutide) or exendin-4 GLP-1 peptides (exenatide and lixisenatide), with different administration frequency (daily or weekly), administration route (oral or subcutaneous injection), and delivery (single or multidose or capsule) [26]. A large body of evidence on GLP-1RAs has demonstrated that these agents are safe (noninferiority to placebo in addition to standard care) and effective (glycemic control), with a minimal risk of hypoglycemic episodes and a significantly reduced risk of major adverse cardiovascular events (MACEs), all-cause mortality, and hospitalization for heart failure following their administration, as well as a slowing of renal dysfunction [2]. The results from the main trials evaluating GLP-1RAs effects are reported in Table 1; all studies were designed to assess noninferiority to placebo in terms of CV safety and superiority to placebo in terms of efficacy (except for REWIND, designed to evaluate superiority to placebo in terms of CV safety). The follow-up duration of these studies was variable, ranging from 16 months in the PIONEER-6 trial to 8.6 years in the SUSTAIN- 6 trial.

However, it is important to remember that not all the GLP-1RAs have demonstrated clear and comparable cardiovascular benefits, which may vary in association with the specific molecule and the current setting of application. In this context, a meta-analysis (14 RCTs, 1262 subjects) of overweight/obese T2D adolescents (<18 years) demonstrated that GLP-1RAs reduced glycated haemoglobin (HbA1c) and fasting plasma glucose (FPG) and induced weight loss (liraglutide superior to exenatide in terms of glucose reduction, exenatide more efficacious than liraglutide in terms of weight loss) [34]. Another meta-analysis conducted on adolescents with overweight/obesity without T2D reported that GLP-1RAs had a better anti-obesity effect when compared to placebo or lifestyle modification (semaglutide is better than liraglutide and exenatide for weight control) [35].

There are also some contraindications, which include a history of pancreatitis or pancreatitis beginning under these treatments, severe gastrointestinal diseases, hypersensitivity and pregnancy, and personal/family history of multiple endocrine neoplasia 2A, 2B, or medullary thyroid cancer [36]. Moreover, adverse effects like gastrointestinal effects (e.g., nausea, vomiting, diarrhea, dyspepsia), mild tachycardia, infections, and headaches may occur. At present, combination with DPP-4 inhibitors is not recommended, despite the significant glycemic improvement, in view of the enhanced hypoglycemic effects. Possible interactions with other anti-diabetic drugs need to be verified [36].

## 4. GLP-1 and GLP-1RAs Effects at the Brain Level

Experimental studies on pharmacological GLP-1R blockade or selective genetic disruption of GLP-1R signaling evidence the key role of GLP-1R signaling for food intake; in particular, one study reported the mechanism by which nucleus-tractus-solitarius-derived GLP-1 modulates neuronal activity and food intake behavior (through the following steps: GLP-1R signaling stimulates the PKA signaling pathway, which promotes phosphorylation of GluA1 S845, increasing GluA1 membrane trafficking and postsynaptic excitatory synaptic transmission), whereas alterations in the paraventricular nucleus of the hypothalamus GLP-1 signaling induced obesity [37]. More recent data highlight the role of GLP-1Rs in the lateral septum, as their targeted knockdown reduces liraglutide’s ability to inhibit feeding and reduces body weight, evidencing the role of these neurons in the control of energy homeostasis and in the modulation of liraglutide effects [38].

Interestingly, intracerebroventricular (ICV) infusion of native GLP-1 increases brown adipose tissue (BAT) thermogenesis (and also browning of white fat), promoting a reduction in food intake and body weight through the activation of hypothalamic neurons [39].

GLP-1RAs (e.g., exenatide and liraglutide) appear effective in modulating BAT thermogenesis and browning, controlling food intake, and increasing energy expenditure through the action on the hypothalamic AMPK pathway, with clinically significant effects; therefore, they are very promising in the obesity setting [40]. Another relevant potential application of GLP-1RAs is emerging for the treatment of neurodegenerative diseases, in view of their neuroprotective properties [41,42].

## 5. DPP-4 and DPP-4is

In humans, the dipeptidyl peptidase-4 (DPP-4) gene is located on chromosome 2q24.3 (spanning 82.3 kb and consisting of 26 exons) [43,44,45]. DPP-4 is a type II transmembrane glycoprotein (766 residues), involved in numerous signaling pathways (e.g., cell–cell interaction and activation of transduction pathways of intracellular signals), although, under some stimuli (e.g., tumor necrosis factor α (TNFα) and chronic low-grade inflammation), this molecule can be released in the blood as a soluble form (sDPP-4, a protein of 727 amino acids) [45,46]. This enzyme cleaves N-terminal dipeptides from incretin hormones (GLP-1 and glucose-dependent insulinotropic polypeptide, GIP), beyond a number of other substrates (e.g., cytokines, growth factors, neuropeptides) [47]. DPP-4 expression has been detected in several tissues and organs (e.g., lungs, small intestine, spleen, liver, kidney, pancreas, endothelial cells, hematopoietic cells). Although sDPP-4 is derived from endothelial cells, epithelial cells, and leukocytes, a high amount of sDPP-4 is released from adipocytes, particularly those present in visceral adipose tissue (with adverse consequences on insulin signaling and sensitivity); therefore, DPP-4 has been identified as an adipokine with diverse para- and endocrine properties. In particular, sDPP-4 has been considered a key factor connecting obesity with inflammation and insulin resistance; DPP-4 regulates adipokines, as leptin and adiponectin, and a negative correlation between blood DPP-4 activity and circulating adiponectin levels in lean and obese subjects has been observed [48,49]. Obesity is associated with increased sDPP-4 levels, which also leads to adipocyte insulin resistance through mechanisms related to the interactions with caveolin-1 (Cav-1), expressed on the cell surface of adipocytes and adipose tissue macrophages, as well as increased hepatic insulin resistance [50]. Moreover, DPP-4 negatively affects the function and the survival of β cells, favors the onset and development of T2D, and has been found to increase in T2D patients [51]. Data obtained in human primary adipocytes treated with short interfering RNA, which induced DPP-4 silencing, showed improved adipocyte insulin sensitivity (by activation of Akt) [52]. Accordingly, models in animals lacking DPP-4 underline the importance of this factor in the regulation of glucose metabolism: in fact, DPP-4-deficient rats showed better glucose tolerance and higher insulin and GLP-1 levels than controls [51]. The interest raised by this molecule is linked to the approval of DPP-4 inhibitors for T2D treatment (gliptins as vildagliptin, saxagliptin, alogliptin, sitagliptin, and linagliptin), as monotherapy or as an adjunctive agent with other drugs, with numerous advantages with respect to traditional treatments [53]. In fact, DPP-4 inhibitors prevent the degradation of GLP-1 and GIP and increase the insulin action after an oral glucose load (incretin action), thus increasing insulin release by the pancreatic β cells, also acting on the α cells to reduce glucagon release and hepatic glucose production [54].

Interestingly, the DPP-4 level has been found to correlate with levels of advanced glycation end products (AGEs), derived from nonenzymatic reaction between reducing sugars and amino groups of proteins, lipids, and nucleic acids, and related to chronic hyperglycemia and uncontrolled T2D. Upon AGEs binding to their receptor (RAGE), the activation of NF-κB occurs, leading to an increase in inflammatory cytokines and oxidative stress [55]. Recent data suggested that AGEs significantly increased DPP-4 expression and sDPP-4 release by tubular cells, whereas AGE and RAGE expression levels are reduced in the kidney of DPP-4-deficient diabetic rats, suggesting the existence of a cross-talk between the AGE–RAGE axis and DPP-4 in the pathogenesis and progression of T2D [56,57]. However, DPP-4 affects many other pathophysiological pathways, not only limited to the regulation of glucose homeostasis and T2D, with multiple effects on inflammation, oxidative stress, and immune response, retaining a key role in the pathogenesis of cardiovascular disease (Table 2). In fact, experimental data suggested that sDPP-4 acts as a pro-inflammatory adipokine (e.g., through the TLR pathway), while DPP-4 inhibition (with either vildagliptin, an enzymatic inhibitor, or mannose-6-phosphate, a competitive binding inhibitor) improves the inflammatory milieu [58]. At the endothelial level, DPP-4 effects appear mediated by both GLP-1-dependent and independent mechanisms [59]. In particular, sDPP-4 acts as a direct mediator of endothelial dysfunction, probably through PAR2 activation and the induction of vasoconstrictor prostanoid release (e.g., thromboxane A2) [60]. Interestingly, DPP-4 inhibition increases circulating endothelial progenitor cells, likely targeting the chemokine stromal-cell-derived factor 1α, an important protective system [61].

In the cardiomyocytes, H2O2 exposure induced DPP-4 expression, whereas knocking down DPP-4 decreased the loss of cell viability, promoting mitochondrial bioenergy and intracellular reactive oxygen species (ROS) production, as well as apoptosis-associated protein expression [62]. However, recent experimental data in mice evidenced that sDPP-4 levels were not associated with inflammation [63]. Moreover, in humans, sDPP-4 appeared to increase obesity and insulin resistance, but it was not associated with systemic metabolic inflammation (IL-6 or CRP levels), evidencing the complexity of the role of sDPP-4 in the inflammatory processes [64].

Targeted inhibition of DPP-4 reduces angiotensin (Ang) II elevation, whereas inhibitors of the angiotensin I-converting enzyme (ACE) or Ang II type 1 receptor (AT1R) blockers downregulate DPP-4 activity in experimental models of cardiometabolic diseases, evidencing a bidirectional relationship between DPP-4 and Ang II [65]. At the renal level, the sDPP-4/TGFBR/SMAD axis may induce epithelial–mesenchymal transition in epithelial cells, which lose epithelial function and characteristics and may promote renal fibrosis [66]. In addition, some experimental studies suggested a relationship between elevated DPP-4 and an unfavorable lipid profile; in humans, an association between sDPP-4 and triglyceride levels was evidenced, while patients treated for 24 months with a DPP-4is-based combination with metformin exhibited an improved lipid profile, especially for triglycerides and LDL, when compared to baseline in T2D patients [67].

For all these effects, different benefits have been attributed to DPP-4is (including improvement of oxidative stress, inflammation, fibrosis and apoptosis, endothelial function, and tissue reparation), related to renal protection and a variety of cardiovascular and metabolic clinical conditions beyond T2D, including hypertension, calcified aortic valve disease, atherosclerosis, and heart failure (HF) [47,68]. In addition, hypoxia modulates DPP-4 expression at the vascular level, although the exact pathophysiological role of DPP-4 in the vasculature is to be further elucidated [69]. Experimental data suggested that DPP-4 inhibition may have direct protective effects on the heart after myocardial infarction by improving cardiac function and decreasing the infarct size; in this context, DPP-4 inhibition induces an antiapoptotic effect, with the involvement of the SDF-1α/CXCR4-mediated STAT3 signaling pathway [70].

Interestingly, DPP-4 activity was associated with risks of major adverse cardiac or cerebrovascular events (cardiovascular mortality, myocardial infarction, heart failure-HF readmission, stroke, non-cardiovascular mortality, and repeated revascularization) in T2D patients with ST-segment elevation myocardial infarction (STEMI) [71]. A positive relationship between circulating DPP-4 activity and the presence and degree of chronic hepatic conditions has been evidenced, including hepatitis C virus (HCV)-related hepatitis, nonalcoholic steatohepatitis (NASH), nonalcoholic fatty liver disease (NAFLD), and progression to cirrhosis; a positive correlation between liver biomarkers alanine aminotransferase (ALT) and aspartate aminotransferase (AST) and DPP-4 activity has been observed in type 1 diabetes [72,73,74,75].

Through its binding with the adenosine deaminase (ADA), this molecule can contribute to autoimmune diseases and cancers, conditions where serum DPP-4 activity and sDPP-4 levels are found altered [76,77,78]. Specifically, it is known that DPP-4 is expressed in different types of immune cells (e.g., CD4(+) and CD8(+) T cells, B cells, macrophages, natural killer and dendritic cells) and regulates the activation of these cells [79]. Nonetheless, although many experimental and clinical findings indicate that DPP-4 plays a role in the pathogenesis of autoimmune diseases, DPP-4 inhibition has controversial effects in autoimmune or inflammatory diseases, which might be related to the ubiquitous tissue distribution and tissue-specific regulation of DPP-4/CD26, as well as to the numerous functions and effects of DPP-4/CD26 [46]. In this context, data obtained from experimental studies are often inconsistent when compared with those conducted on humans, and many aspects require deeper investigations (e.g., role of DPP-4 in different cell types, temporal and spatial characteristics of DPP-4 expression across different disease stages, identification of possible ligands for DPP-4, and approaches for targeting nonenzymatic DPP-4 activity) [46,80].

DPP-4is, also known as gliptins, include sitagliptin, saxagliptin, linagliptin, alogliptin, and vildagliptin [81]. The adverse effects of DPP-4is are mainly related to gastrointestinal tract discomforts (e.g., nausea, diarrhea), pancreatitis, and skin reactions (e.g., rash, angioedema, pruritus) [82]. Contraindications include a history of pancreatitis or diabetic ketoacidosis, hypoglycemia, and angioedema [81].

## 6. SGLT2 and SGLT2is

**Figure 2:**
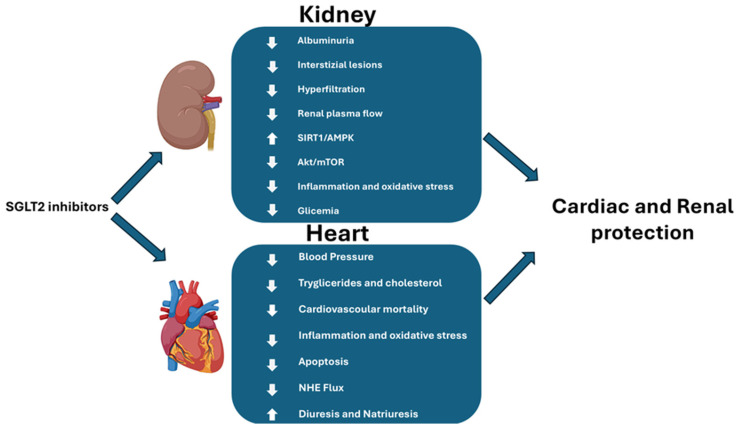
Effect of SGLT2 inhibitors at renal and cardiac levels.

The sodium-glucose cotransporter 2 (SGLT2) is located in the apical membrane of the S1 and S2 segments of the proximal tubule and is responsible for 90% of glucose and sodium reabsorption [83]. In physiological conditions, the tubuloglomerular feedback (TGF) signaling can adjust a stable glomerular filtration rate (GFR) by modulation of preglomerular arteriole tone. Chronic hyperglycemia increases proximal SGLT2-mediated reabsorption of sodium (Na+); glucose impairs this feedback mechanism, and the macula densa is exposed to lowered sodium concentrations, despite increased GFR, leading to the impairment of TGF signaling with a consequent increase in blood flow into the glomerulus; thus, glomerular pressure increases and leads to glomerular hyperfiltration until organ damage develops [84]. The pathophysiology of diabetic nephropathy is mainly due to the enhanced glucose uptake in tubule cells via SGLT2 inducing oxidative stress, expression of inflammatory and fibrosis genes, and increased apoptosis (Figure 2) [85,86].

The inhibition of SGLT2 with empagliflozin blocks proximal tubule sodium and glucose reabsorption, leading to increased sodium delivery to the macula densa; this condition restores TGF via appropriate modulation of arteriolar tone (e.g., afferent vasoconstriction), which in turn reduces renal plasma flow and hyperfiltration. Histological evidence of nephropathy was attenuated using dapagliflozin (another SGLT2 inhibitor), which reduces gene expression of inflammation and oxidative stress in the kidney of db/db mice. Moreover, dapagliflozin suppresses the high-glucose-induced gene expression of inflammatory cytokines and oxidative stress in cultured proximal tubular epithelial (mProx24) cells [87].

The expression and activity of SGLT2 increased in subjects with T2D [88]. The SGLT2i mechanism of action is independent of insulin secretion by pancreatic β cells or insulin resistance in the body; SGLT2is compete with the SGLT2 protein for glucose binding in the renal tubules, causing an increase in glucose excretion, sodium, and water in urine and a decrease in blood glucose and reduced volume load [89,90,91]. In human subjects affected by diabetic nephropathy, the expression of SGLT2 mRNA and protein is increased in renal biopsies; moreover, in db/db mice, SGLT2 inhibition modulates renal lipid metabolism and inflammation, counteracting the development of nephropathy [88]. Moreover, the SGLT2i empagliflozin prevented the increase in inflammation and fibrosis induced by high glucose by blocking glucose transport, without inducing a compensatory increase in SGLT1/GLUT2 expression in the human kidney proximal tubular cell line (HK2 cells) [92].

Canagliflozin (SGLT2i) contributes to decreased inflammation, extracellular matrix turnover, and fibrosis (300 mg/day decreased plasma levels of TNF receptor 1, IL-6, matrix metalloproteinase 7, and fibronectin 1 after 2 years of follow-up) [93]. SGLT2is promote TGF and decrease glomerular hyperfiltration [94].

In a model of non-proteinuric diabetic kidney disease of high-fat-diet-fed ApoE-knockout mice, ATP production changes from lipolysis- to ketolysis-dependent, due to hyperactivation of the mechanistic target of rapamycin complex 1 (mTORC1); in this study, empagliflozin raised the endogenous levels of ketone body (KB) and prevented a reduction in renal ATP levels and organ damage in the mice, an effect abolished by gene deletion of Hmgcs2, a rate-limiting enzyme of ketogenesis [95]. These results suggested that SGLT2is promote renoprotection through KB elevation that in turn corrects mTORC1 hyperactivation occurring in non-proteinuric and proteinuric diabetic kidney disease.

SGLT2is reduce oxidative stress by activating SIRT1/AMPK and inhibiting Akt/mTOR, thus collectively contributing to improved cellular energy efficiency, increased resistance to oxidative damage, and ultimately cardio- and renoprotection (reduced blood volume and blood pressure, promotion of diuresis and natriuresis, and glycosuria), especially beneficial in HF cases where excessive fluid can place additional strain on the heart [96,97]. Marfella et al. investigated SGLT2 expression in human hearts of diabetic and non-diabetic patients, evidencing that the expression of SGLT2 in the explanted heart of diabetics was increased when compared to non-diabetic patients, whereas the presence of SGLT2 in cardiomyocytes accounts for the effects of SGLT2is on metabolic pathways within cardiomyocytes [98]. These drugs may also exert other effects to prevent the severity of HF (Figure 2).

Many trials were designed to explore cardiovascular safety or benefit using SGLT2i molecules, including empagliflozin, dapagliflozin, canagliflozin, and ertugliflozin, which greatly differ in their characteristics, including the number of participants, study duration, the inclusion of patients with HF or renal disease, HF characterized by reduced ejection fraction (HFrEF) or preserved ejection fraction (HFpEF), and associated comorbidities or risk factors, clearly implying differences in the risk and rate of cardiovascular or renal events recorded during the investigations [2]. The beneficial effects of these molecules at cardiovascular and renal levels are multiple and ultimately include a natriuretic effect (with reduced risk of hospitalizations for HF and the renal damage slowdown); improvement of the lipid profile, inflammation, oxidative stress, endothelial function, and blood pressure; body weight loss; and reduced risk of MACEs, cardiovascular mortality, and HF hospitalization in high-cardiovascular-risk T2D patients [2,99,100].

SGLT2is should not be used in pregnant women, patients with renal glomerular filtration <20 mL/min/1.73 m^2^, or patients with diabetic ketoacidosis. Adverse effects may occur, such as an increased risk of non-sexually transmitted genital infections and/or urinary infections, an increased risk of hypotension, and in rare cases, diabetic ketoacidosis [101].

SGLT2is have shown reliable benefits in reducing cardiovascular mortality; in particular, the treatment with empagliflozin in the EMPA-REG OUTCOME study (T2D patients who were at high risk of cardiovascular disease) showed no significant between-group differences in the rates of myocardial infarction or stroke, although in the empagliflozin group, significantly lower rates of death from cardiovascular causes (3.7% vs. 5.9% in the placebo group; 38% relative risk reduction), hospitalization for HF (2.7% and 4.1%, respectively; 35% relative risk reduction), and death from any cause (5.7% and 8.3%, respectively; 32% relative risk reduction) were observed [102]. The CANVAS trial confirmed the same result with the use of canagliflozin [103], as did the DECLARE TIMI trial where dapagliflozin was used [104].

Treatment with empagliflozin in patients with T2D improves cardiac efficiency and energy by increasing circulating ketone concentration, thus representing a “sparing” fuel for the heart [105,106,107]. SGLT2is shift the heart’s energy metabolism toward more efficient substrates like KB, which improve myocardial energy efficiency and reduce stress on heart muscle cells, upregulating the fatty acid metabolism [108,109].

HF severity is characterized by the increase in inflammation [108], where the use of SGLT2is increases the circulating levels of ß-hydroxybutyrate, which is an effective blocker of the NLRP3 inflammasome-mediated inflammatory process, which plays an important role in mediating inflammation [110,111]. The incubation of human atrial cardiomyocytes with canagliflozin reduced inflammation and apoptosis, suppressing myocardial NADPH oxidase activity, and improved nitric oxide synthase (NOS) coupling via SGLT1/AMPK/Rac1 signaling [112]. Moreover, both empagliflozin and canagliflozin lower the levels of AGE products, downregulating the AGE–RAGE axis, which results in lowered inflammation and increased cardiac protection [113,114].

Na^+^ and Ca^2+^ accumulation in the heart, due to the increase in Na+/H+ exchanger (NHE), could cause a failing heart. The capacity to develop oxidative stress, arrhythmias, and HF are all associated with, and at least partly driven by, intracellular cardiomyocyte Na^+^ and Ca^2+^ concentrations [115,116,117]. Experimental studies suggested that the use of SGLT2 inhibited NHE and protected HF; when cardiac cytoplasmic Na^+^ and Ca^2+^ concentrations and mitochondrial Ca^2+^ were measured fluorometrically in isolated ventricular myocytes from rabbits and rats, the results evidenced that empagliflozin directly inhibited NHE flux, caused by a reduction in cytoplasmic Na+ and Ca^2+^ concentrations and an increase in mitochondrial Ca^2^ [118]. The structure and integrity of mitochondria are essential for the physiological function of the heart; in this context, the effect of empagliflozin on mitochondrial ultrastructure after cardiac arrest (CA) was examined by transmission electron microscopy. Mitochondria appeared smaller in the CA and vehicle group compared with mitochondria from the sham group, suggesting mitochondrial fission after CA. Treatment with empagliflozin significantly decreased mitochondrial area after CA and reduced the expression of Drp1p, a protein that regulates mitochondrial fission. In cardiomyocytes after CA, the number of inter-mitochondrial junctions (an index of highly active mitochondria) was significantly decreased, while empagliflozin treatment significantly increased the number of inter-mitochondrial junctions. Modulating mitochondrial bioenergetics may be an effective treatment for HF since empagliflozin treatment significantly increased the myocardial ATP levels, myocardial mitochondrial complex I activity, and myocardial mitochondrial respiratory control ratio at 6 and 24 h after return of spontaneous circulation (ROSC) [119]. In a rat model of insulin resistance and obesity, empagliflozin regulates mitochondrial dynamics, increasing fusion protein mitofusin-2 (MFN2) expression, while decreasing the levels of the fission protein dynamin-related protein 1 (DRP1) in white adipose tissue [120]. In T2D mice, empagliflozin restores AMP/ATP activation of AMPK, inducing Drp1S637 phosphorylation and decreasing Drp1S616 phosphorylation, which leads to the inhibition of mitochondrial fission, improving cardiac microvascular injury [121].

When sotaglifozin was administered (30 mg/kg/d for 6 weeks) in obese rats, with metabolic-syndrome-related models of HF with HFpEF, and wild-type rats, this treatment ameliorated left atrial enlargement in HFpEF in vivo, prevented mitochondrial swelling, enhanced mitochondrial Ca^2+^ buffer capacity in HFpEF, improved mitochondrial fission and reactive oxygen species (ROS) production during glucose starvation, and averted Ca^2+^ accumulation upon glycolytic inhibition [122]. Other data suggested that dapagliflozin improved mitochondrial dynamics optic atrophy 1 (OPA1), DRP1, and MFN2, together with the levels of proteins related to skeletal muscle mitochondrial biogenesis (PGC-1α, NRF1, TFAM, and COX IV) and mitophagy (PGAM5 and PINK1) in T2D rats [123], whereas it induced improvement of fusion–fission proteins mitofusin-1 (Mfn-1,) Mfn-2, and mitochondrial fission 1 (Fis-1) in a rat model of metabolic syndrome [124].

## 7. New Drugs, Comparative Analysis, and Combination/Multidrug Therapy

Other new drugs are under study, such as new oral GLP-1RAs (e.g., danuglipron or orforglipron) or dual glucose-dependent insulinotropic polypeptide (GIP) and glucagon-like peptide-1 (GLP-1) receptor agonist or triple agonists, glucagon-like peptide 1 (GLP-1), glucose-dependent insulinotropic polypeptide (GIP), and glucagon agonists (retatrutide-LY3437943), which can expand the arsenal of treatment opportunities in the future. A meta-analysis including four RCTs (published 2021–2023) evidenced that danuglipron is reliable for glycemic control and weight reduction in T2D patients; nonetheless, adverse outcomes (e.g., diarrhea, nausea, vomiting, and decreased appetite, with dose-related effects) were observed [125]. A recent meta-analysis, including seven RCTs (1037 patients), evaluated the efficacy and safety of orforglipron and danuglipron in subjects with obesity, T2D, or both. When compared to controls, the two drugs efficiently reduced glycemia and weight in T2D, obesity, or both; however, more adverse effects (e.g., gastrointestinal) were recorded [126]. A following meta-analysis investigated the effects of once-daily oral orforglipron on weight and metabolic markers in adults; the results showed the efficacy of this drug on body weight, as compared with placebo, although an increase in non-severe gastrointestinal adverse events was also observed [127]. Especially in view of the frequent adverse effects leading to discontinuation, more knowledge on efficacy, safety, and tolerability is surely needed before their introduction in the clinical practice against T2D or obesity.

Tirzepatide, a dual GIP and GLP-1 receptor agonist, administered once a week subcutaneously showed the properties of controlling glycemia and improving insulin sensitivity and the lipid profile, as well as reducing weight, without increased risk of hypoglycemia and with a safety profile similar to that of GLP-1RAs [128]. LY3437943 is a triple agonist peptide that combines activity at the glucagon, GIP, and GLP-1 receptors [129]. In a phase 1b study, LY3437943, administered once a week, demonstrated an acceptable safety profile, together with significant glucose and weight lowering in subjects with T2D [130]. In adults with obesity, LY3437943 treatment for 48 weeks induced significant body weight lowering; the most common adverse effects were gastrointestinal, dose-related, mild–moderate, and reduced in part by a lower starting dose (2 mg vs. 4 mg) [131]. 

Certainly, these compounds may represent new promising and appealing therapeutic tools, although at present, it is necessary to better evaluate their long-term effects and determine their potential cardiometabolic benefits [132].

Currently, combination/multiple-drug therapy is becoming more popular than monotherapy. A very large study focused on the risk of cardiovascular events in 1.5 million T2D patients initiating a second-line hypoglycemic therapy after metformin; SGLT2is and GLP-1RAs were associated with a lower risk of adverse outcomes when compared to DPP-4is [133]. A meta-analysis comparing compounds belonging to the classes of GLP-1RAs, SGLT2is, and DPP-4is was performed on T2D subjects with low and high risk for CV disease (including those with established CV disease and/or kidney disease); metformin was generally the background therapy for the majority of subjects. The results obtained suggested that SGLT2is and GLP-1RAs are more effective on mortality and major adverse cardiovascular events (MACEs) compared to DPP-4is. Moreover, SGLT2is are more beneficial in patients with HF and kidney disease. Overall, the effects of SGLT-2 inhibitors and GLP-1RAs were significant, especially in patients with known or high risk of CV disease, but more limited for the low-risk subgroup [134]. An analysis of nine observational studies showed a significant reduction in CV and renal outcomes with the GLP-1RA/SGLT2i combination, compared with GLP-1RA and SGLT2i monotherapy [135]. These findings were confirmed by a further analysis including 5 RCTs, 10 post hoc analyses, and 1 observational study, where the combination therapy reduced the risk of cardiorenal outcomes, HF-related outcomes, and all-cause mortality when compared with monotherapy of either agent [136]. A recent meta-analysis conducted on elderly T2D patients (15 studies, *n* = 11,679) evidenced that a combination of GLP-1RAs with SGLT2is (with an intervention period of at least six months), beyond demonstrating a positive safety profile, gave more benefits over monotherapy in terms of glycemic control, weight management, and CV protection [137]. Another meta-analysis reported that the combination of SGLT2is and GLP-1RAs reduced HbA1c, FPG, and systolic blood pressure (SBP) without increasing hypoglycemia risk, compared with SGLT2i monotherapy, but not compared to GLP-1RA treatment alone [138].

Clearly, the combined therapy may permit a faster and easier achievement of therapeutic targets by involving more complementary drugs targeting different pathogenic mechanisms [139]. However, it also retains disadvantages: e.g., possible low adherence due to difficulties related to adhering to a multidrug regimen (more drugs, more doses, to be taken at different times) and higher costs (although long-term cost implications with respect to the gain in disease delay of combination therapy have not yet been carried out, some analyses suggest that initial higher costs may be partially offset by a decrease in T2D-related complications and a delayed insulin starting point) [140].

## 8. Comparison Between SGLT2is, GLP-1RAs, and DPP-4is

Currently, treating T2D is becoming increasingly important in view of the epidemiological evolution of the disease, characterized by its progressive increase in prevalence and incidence globally (especially in developed world areas), earlier age of disease onset (in subjects younger than 45 years, often with obesity), and higher morbidity and mortality in patients who develop the disease at a younger age [141]. However, the arsenal of therapeutic options, extended in recent years to SGLT2is, GLP-1RAs, and DPP-4is, has overturned the approach to the management of T2D, moving from a conventional, simplistic, glucose-centered point of view to a holistic, cardiorenal–metabolic, and multi-target organ-protective perspective. As discussed above, available results have shown that these new pharmacological options, in addition to their anti-hyperglycemic effect, present many cardiorenal and metabolic effects, which can clearly differ according to the timing, dose, and mode of administration, the type and actions of the drugs, and the degree of target organ protection and weight reduction achieved. As an example, the results of a meta-analysis evaluating the risk of cardiorenal effects exerted by GLP-1RAs and SGLT2is evidenced similar benefits (about 12–14% risk reduction), although GLP-1RAs are superior for stroke risk reduction [142]. Instead, SGLT2is are more effective in the setting of HF and in patients with chronic kidney disease (with or without T2D) for the treatment and prevention of these conditions, whereas GLP-1RAs appear to remain more compelling in weight loss, glycemic control, and cardiovascular benefits in T2D patients [143]. These conclusions were confirmed by data from a recent meta-analysis of nine large trials, which evidenced that GLP-1RAs showed a significant reduction in MACEs (RR = 0.92; 95%CI 0.87–0.97), CV death (0.88; 0.80–0.97), and overall death (0.89; 0.82–0.96), whereas SGLT2is showed a significant reduction in HF hospitalization (0.72; 0.6–0.86) compared to placebo. Importantly, ongoing studies are now deepening the understanding of the effects of GLP-1RAs in different HF cohorts (e.g., patients with HFpEF and those with advanced HF) [144,145,146].

It should be noted that some clinical trials have reported inconsistencies in CV benefits, particularly regarding DPP-4is (e.g., saxagliptin and its association with HF risk) [147]. In particular, the SAVOR-TIMI 53 (Saxagliptin Assessment of Vascular Outcomes Recorded in Patients with Diabetes Mellitus) clinical trial evidenced neutral effects of saxagliptin in terms of cardiovascular outcomes, but an increased risk of hospitalization for HF, especially in patients with high levels of natriuretic peptides, previous HF, or chronic kidney disease [148]. Experimental data suggest that saxagliptin and its substrate neuropeptide Y may interact with cardiac remodeling, acting on pathophysiological mechanisms of end-stage chronic HF. In fact, the DPP4 enzyme could be compensatory against the altered neuropeptide tone provoked by the high sympathetic activity in HF. Instead, the inhibition of DPP4 by saxagliptin could reduce this adaptive response to cardiac remodeling, increasing myocardial damage [149].

A recent review analyzed the role of DPP-4is in patients with T2D and HF, highlighting some controversial results, since some data indicate an increased risk of HF hospitalizations with specific DPP-4is (e.g., saxagliptin), while others report neutral effects, which could indicate effects more related to specific individual molecular characteristics than to a generic common effect of the entire class of drugs [150]. Accordingly, a meta-analysis (54 studies with 74,737 participants) showed that DPP-4is were not associated with an increased risk of HF when considered as a class; when analyzed individually, saxagliptin was significantly associated with the increased risk of HF (RR 1.215; 95 CI, 1.028–1.437; *p* = 0.022), especially in patients at high CV risk (age ≥ 65 years, T2D duration ≥ 10 years and BMI ≥ 30 kg/m^2^), but not the other DPP-4is, suggesting a differential effect of each DPP-4 inhibitor on the risk of HF [151]. This conclusion was further confirmed by a more recent meta-analysis reporting that DPP-4is as a class are neutral in terms of MACEs, all-cause mortality, and HF, although saxagliptin appears associated with an increased risk of hospitalization for HF [152]. Another meta-analysis (50 randomized controlled trials) performed to assess the association between DPP-4is and the risk of HF in T2D patients evidenced that vildagliptin appears to be the safest option with regard to the risk of HF, followed by saxagliptin, sitagliptin, linagliptin, and alogliptin [153].

Clearly, these observations must be taken into account when prescribing DDP-4is, always with a view to a targeted therapy that is increasingly designed to meet the specific needs of the individual patient.

## 9. Conclusions

Future studies will provide further insights to refine the role of these new drugs, continuing to expand their utility beyond T2D, to treat renal–cardiometabolic conditions in a more integrated approach as a single entity to target. Additional agents are being studied with the aim of improving care in the future, allowing for more targeted and appropriate treatment with even greater potential for the individualization of therapy. Importantly, further research on long-term therapy should verify the long-term efficacy, other possible pleiotropic effects of each drug, and the safety profile of combination therapy in order to reveal broader clinical applicability that will need to be validated in different patient populations.

## Figures and Tables

**Table 1:** Main CardioVascular Outcome Trials (CVOTs) on GLP-1RAs in T2D patients.

Drug	Study Acronym	Patient Number	Dose	Frequency	Primary CV Outcome	Results vs. Placebo	Ref
Exenatide	EXCEL	14752	2 mg	O/W	CV death, AMI, stroke	noninferiority	[27]
Lixisenatide	ELIXA	6068	20 µg	O/D	CV death, AMI, stroke	noninferiority	[28]
Liraglutide	LEADER	9340	1.8 (or MTD)	O/D	CV death, AMI, stroke	superiority	[29]
Semaglutide	SUSTAIN-6	2735	0.5–1	O/W	CV death, AMI, stroke	superiority	[30]
Oral Semaglutide	PIONEER-6	3183	3–14	O/D	CV death, AMI, stroke	noninferiority	[31]
Albiglutide	HARMONY OUTCOMES	9.463	30 (until 50)	O/W	CV death, AMI, stroke	superiority	[32]
Dulaglutide	REWIND	9901	1.5	O/W	CV death, AMI, stroke	superiority	[33]

O/D = once/daily, O/W = once/weekly, MTD = maximum tolerated dose.

**Table 2:** Potential adverse effects of DPP-4 on target cells, tissues, and systems.

Adipose tissue	Inflammation Fibrosis VAT insulin resistance
Pancreatic islets	β-cell function and survival reduction Insulin secretion impairment
Liver	Inflammation Altered lipid metabolism Insulin resistance Steatosis/fibrosis
T lymphocytes	Immune response
Macrophages	Inflammation Chemotaxis
NK cells, eosinophils, basophils	Inflammation
Kidney	Inflammation/oxidative stress Fibrosis
CVD	Endothelial progenitor cell reduction Endothelial dysfunction/impaired vasodilation Inflammation/oxidative stress Myocardial fibrosis Reduced cardiac function

VAT = visceral adipose tissue, CVD = cardiovascular disease.

## Data Availability

No new data were created or analyzed in this study.

## Abbreviations

The following abbreviations are used in this manuscript:

T2D	type 2 diabetes
GLP-1	glucagon-like peptide 1
SGLT2	sodium-glucose cotransporter 2
DPP-4	dipeptidyl peptidase-4
NTS	nucleus of the solitary tract
GLP-1RA	GLP-1 receptor agonist
GLP-1R	GLP-1 receptor
cAMP	cyclic adenosine monophosphate
PKA	protein kinase A
cAMP-GEFII or Epac2	cAMP-regulated guanine nucleotide exchange factor
CREB	cAMP-responsive element binding
MACE	major adverse cardiovascular event
CVOTs	CardioVascular Outcome Trials
ICV	intracerebroventricular
BAT	brown adipose tissue
DPP-4i	dipeptidyl peptidase 4 inhibitor
sDPP-4	dipeptidyl peptidase 4 soluble
AGEs	advanced glycation end products
RAGE	advanced glycation end product receptor
ROS	reactive oxygen species
Ang	angiotensin
ACE	angiotensin I-converting enzyme
AT1R	Ang II type 1 receptor
HF	heart failure
STEMI	ST-segment elevation myocardial infarction
HCV	hepatitis C virus
ALT	alanine aminotransferase
AST	aspartate aminotransferase
ADA	adenosine deaminase
TGF	tubuloglomerular feedback
GFR	glomerular filtration rate
mProx24	proximal tubular epithelial cells
SGLT2is	SGLT2 inhibitors
HK2 cells	human kidney proximal tubular cell line
mTORC1	rapamycin complex 1
KB	ketone body
HFrEF	reduced ejection fraction
HFpEF	preserved ejection fraction
NOS	nitric oxide synthase
NHE	Na+/H+ exchanger CA
CA	cardiac arrest
ROSC	return of spontaneous circulation
MFN	mitofusin
DRP1	dynamin-related protein 1
AMP	adenosine monophosphate
AMPK	activated protein kinase
OPA1	optic atrophy 1
Fis-1	fission 1
GIP	glucose-dependent insulinotropic polypeptide
